# Pseudo-nullclines enable the analysis and prediction of signaling model dynamics

**DOI:** 10.3389/fcell.2023.1209589

**Published:** 2023-09-28

**Authors:** Juan Ignacio Marrone, Jacques-Alexandre Sepulchre, Alejandra C. Ventura

**Affiliations:** ^1^ Universidad de Buenos Aires, Facultad de Ciencias Exactas y Naturales, Departamento de Física. Ciudad Universitaria, Buenos Aires, Argentina; ^2^ CONICET—Universidad de Buenos Aires, Instituto de Fisiología, Biología Molecular y Neurociencias (IFIBYNE). Ciudad Universitaria, Buenos Aires, Argentina; ^3^ Institut de Physique de Nice, CNRS UMR7010, Université Côte d’Azur, Nice, France

**Keywords:** pseudo-nullclines, oscillations, bistability, MAPK, signaling, bifurcations, cell cycle

## Abstract

A powerful method to qualitatively analyze a 2D system is the use of nullclines, curves which separate regions of the plane where the sign of the time derivatives is constant, with their intersections corresponding to steady states. As a quick way to sketch the phase portrait of the system, they can be sufficient to understand the qualitative dynamics at play without integrating the differential equations. While it cannot be extended straightforwardly for dimensions higher than 2, sometimes the phase portrait can still be projected onto a 2-dimensional subspace, with some curves becoming pseudo-nullclines. In this work, we study cell signaling models of dimension higher than 2 with behaviors such as oscillations and bistability. Pseudo-nullclines are defined and used to qualitatively analyze the dynamics involved. Our method applies when a system can be decomposed into 2 modules, mutually coupled through 2 scalar variables. At the same time, it helps track bifurcations in a quick and efficient manner, key for understanding the different behaviors. Our results are both consistent with the expected dynamics, and also lead to new responses like excitability. Further work could test the method for other regions of parameter space and determine how to extend it to three-module systems.

## 1 Introduction

In cell signaling, mathematical modeling plays an important role in analyzing and predicting different systems behavior. The range of complexity is vast, with examples as different as the two-dimensional Fitzhugh–Nagumo model (FitzHugh, 1961) and a description of the MAPK cascade with 23 equations (Kochańczyk et al., 2017).

In general, it is well known that most nonlinear differential equations modeling biological systems are not analytically solvable. Therefore, the goal of qualitative analysis of dynamical systems is to provide information about its possible behaviors without having access to its analytical solutions. In this context, a powerful method to analyze qualitatively a planar (i.e., 2D) system is the use of nullclines. These are curves where the derivative of one of the variables is equal to zero. These curves separate regions of the plane where the sign of the derivatives is constant. Moreover, their intersections correspond to steady states of the dynamics. This information can provide a quick way to sketch the phase portrait of the system, like for instance the aforementioned Fitzhugh–Nagumo model. Thus, the technique of nullclines is sometimes sufficient to understand the qualitative dynamics of the system without integrating their differential equations.

However, this technique cannot be extended straightforwardly to phase spaces of dimension higher than 2 because the geometrical objects corresponding to the nullclines are no longer curves but more generally (hyper-)surfaces of codimension-1. Nevertheless, there are cases where the phase portrait of the system can still be projected onto a 2-dimensional subspace, with some curves playing the role of pseudo-nullclines. When applicable, phase plane analysis, and in particular the concept of nullclines, has been one of the most useful tools for the qualitative analysis of dynamical systems. Since the main limitation of the nullcline method is its restricted application to a 2-dimensional phase space, any extension of said method to a higher number of dimensions should be valuable.

In this work, we study signaling models of dimension higher than 2, where pseudo-nullclines are defined and used to qualitatively analyze the system dynamics. The first one is an early cell cycle model in *Xenopus laevis* embryo (Tsai et al., 2014). The authors study the change in the oscillatory behavior during this developmental phase, which is present across different phyla. The second example we analyze corresponds to a subsystem of the Mitogen Activated Protein Kinase (MAPK) cascade, found in all eucaryotic cells. Signals from growth factors in cell surface receptors activate three sequential levels of proteins, with the output of the cascade responsible for the phosphorylation of multiple transcription factors. This leads to its involvement in responses like proliferation and differentiation (Lewis, et al., 1998; Schaeffer and Weber, 1999; Kochańczyk et al., 2017). The well-studied model by Huang and Ferrell consists of 22 equations describing the three-level cascade (Huang and Ferrell, 1996). The last two levels, corresponding to double phosphorylation (DP) cycles, constitute the motif that we study in this work.

Our method applies when a system can be decomposed into 2 modules which are mutually coupled through 2 scalar variables. We show that, by projecting the whole dynamics onto the subspace subtended by the two scalar variables, we can define curves that play the role of pseudo-nullclines. Intersections of these pseudo-nullclines correspond to steady states of the full system. Although the use of these pseudo-nullclines is more limited than with true nullclines, we show that this approach can be useful to figure out the onset of oscillations, and other dynamical behaviors like excitability, for a system whose actual phase space dimension is larger than 2. Other works use pseudo-nullclines to analyze different cell cycle motifs (Tyson and Novák, 2022), by using specific features only applicable to those models. We propose a more systematic approach based on the modularity of the analyzed systems.

We illustrate that situations where the pseudo-nullclines intersect transversely or tangentially enable the distinction of phase portraits of oscillations described respectively by supercritical Hopf or by SNIC bifurcations, while also pointing toward Saddle-Homoclinic bifurcations. On the other hand, we show that these pseudo-nullclines admit a natural interpretation in terms of response functions of each module submitted to a constant input of the other module.

## 2 Methods

The idea of the method is to decompose the system in 2 modules, assuming that the coupling between the modules is one-dimensional. This means that if the variables of the modules are denoted respectively by two sets of real variables, i.e., $x=\left(x_{1},x_{2},\ldots ,x_{n}\right)$ and $y=\left(y_{1},y_{2},\ldots ,y_{m}\right)$ , the model equations can be written as:

$$\frac{dx}{dt}=f\left(x,\alpha \left(y\right)\right)$$

$$\frac{dy}{dt}=g\left(y,\beta \left(x\right)\right)$$ (1)

where $\alpha \left(y\right)$ and $\beta \left(x\right)$ are two real-valued functions. Such a system can be seen as a first module, described by equations $\frac{dx}{dt}=f\left(x,a\right)$ , where *a* is some input parameter, interconnected with a second module whose equations are $\frac{dy}{dt}=g\left(y,b\right)$ , with *b* being the corresponding input parameter. The interconnection comes from replacing the input *a* of the first module by the function $\alpha \left(y\right)$ , and the input *b* of the second module by $\beta \left(x\right)$ . Decomposing a system into two interconnected modules has been considered in the literature by (Angeli et al., 2004).

To simplify the presentation and the notations in what follows we will continue with a basic example, where the coupling functions are simply $\alpha \left(y\right)=y_{1}$ and $\beta \left(x\right)=x_{1}$ . The extension to a more general function is easy and is included at the end of the Supplementary Material, along with a sketch of the general scheme.

Thus, now a stationary state $\left.{\left(x\right.}^{*},y^{*}\right)$ of system (1) is a solution of the system of equations:

$$f\left(x,y_{1}\right)=0$$

$$g\left(y,x_{1}\right)=0$$

Suppose that the solutions of this system of equations can be written as follows:

$$x=X\left(y_{1}\right)$$

$$y=Y\left(x_{1}\right)$$ (2)

Then, by projecting these functions on the plane of coordinates $\left(x_{1},y_{1}\right)$ , we define pseudo-nullclines of the system as two curves C_1_ and C_2_ whose graphs are respectively given by the parametrizations $\left(X_{1}\left(y_{1}\right),y_{1}\right)$ and $\left(x_{1},Y_{1}\left(x_{1}\right)\right)$ . The first curve can be seen as the response function of (component 1 of) the first module with respect to its input parameter y_1_. Similarly, one can interpret the second pseudo-nullcline as the response function of the second module submitted to its input parameter x_1_. One advantage of this definition is that by construction said stationary states of the couple modules must be found among the intersections of the two pseudo-nullclines. Indeed, by definition $\left.{\left(x\right.}_{1}^{*},y_{1}^{*}\right)$ can be written in two ways, either $\left(X_{1}\left(y_{1}^{*}\right),y_{1}^{*}\right)$ , or $\left.{\left(x\right.}_{1}^{*},Y_{1}\left(x_{1}^{*}\right)\right)$ , thus belonging to the two graphs of C_1_ and C_2_.

Conversely, if $\left.{\left(x\right.}_{1}^{*},y_{1}^{*}\right)$ belongs to the intersection set of the pseudo-nullclines C_1_ and C_2_, then:

$$x_{1}^{*}=X_{1}\left(y_{1}^{*}\right)$$

$$y_{1}^{*}=Y_{1}\left(x_{1}^{*}\right)$$

And by construction, the functions X and Y satisfy the steady state equations:

$$f\left(X\left(y_{1}^{*}\right),y_{1}^{*}\right)=0$$

$${g(Y\left(x_{1}^{*}\right),x}_{1}^{*})=0$$

In other words, $x^{*}=X\left(y_{1}^{*}\right)$ and $y^{*}=Y\left(x_{1}^{*}\right)$ constitute a steady state of the coupled system since they satisfy the system of equations:

$$f\left(x^{*},y_{1}^{*}\right)=0$$

$${g(y^{*},x}_{1}^{*})=0$$

Another advantage of this geometrical method is that it is able to reveal a limit point bifurcation, like a saddle-node bifurcation. As it shown in the Supplementary Material, this occurs when a steady state corresponds to a tangential intersection of the pseudo-nullclines. In particular, this feature enables to distinguish between a SNIC bifurcation or a Hopf bifurcation because in the first case oscillations appear through a tangent bifurcation, whereas in the second case the pseudo-nullclines intersect transversely. Both cases are illustrated by applying our method to different signaling motifs studied in the Results section.

## 3 Results

### 3.1 Pseudo-nullclines method applied to a cell cycle model combining positive and negative feedback loops

**FIGURE 1:**
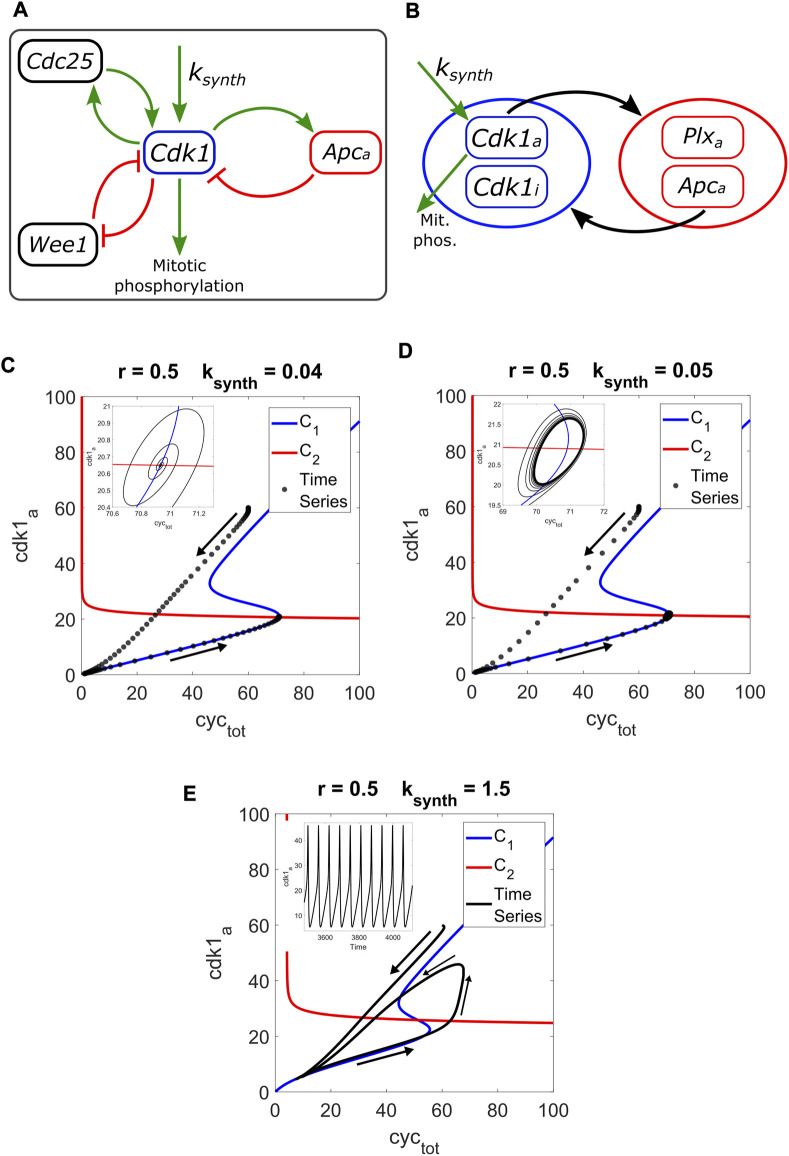
**(A)** Scheme of the cell cycle model, based on the one found in (Tsai et al., 2014). The parameter k_synth_ (synthesis rate of the cyclin) acts as the input of the system. Two positive feedbacks (with Cdc25 and Wee1) and one negative feedback (with Apc_a_) govern the motif. The output Cdk1 (active) is involved in the mitotic phosphorylation. **(B)** Motif scheme based on the pseudo-nullcline method, separating the two modules, and representing how they are interconnected. More details on the equations can be found in the Supplementary Material. **(C)** Pseudo-nullclines C_1_ (in blue) and C_2_ (in red) for r = 0.5 and input = 0.04, with the corresponding time series (in black). Arrows denote the trajectory taken by the system, from cdk1_a_ = 60 to a steady state represented by the curves intersection. **(D)** The input is now 0.05, leading to a small limit cycle around the intersection. **(E)** With input = 1.5, the limit cycle grows in amplitude, following the lower branch of C_1_ but not the upper one, as shown in (Tsai et al., 2008).

In (Tsai et al., 2014), the authors study an oscillatory cell cycle model in *X. laevis* embryos, where the period and shape of the oscillation change between the first mitotic cycle and the subsequent cycles. They analyze the system obtaining experimental data and running computational simulations. A scheme of the model is presented in Figure 1A, showing the two positive feedback loops and the negative one involved.

The system of equations is as follows, written in a more generic manner (see Supplementary Material for the equations in detail):

$$\frac{d\left[{cdk1}_{a}\right]}{dt}=f_{1}\left(\left[{cdk1}_{a}\right],\left[{cdk1}_{i}\right],\left[{apc}_{a}\right]\right)$$

$$\frac{d\left[{cdk1}_{i}\right]}{dt}=f_{2}\left(\left[{cdk1}_{a}\right],\left[{cdk1}_{i}\right],\left[{apc}_{a}\right]\right)$$

$$\frac{d\left[{plx}_{a}\right]}{dt}=g_{1}\left(\left[{plx}_{a}\right],\left[{cdk1}_{a}\right]\right)$$

$$\frac{d\left[{apc}_{a}\right]}{dt}=g_{2}\left(\left[{apc}_{a}\right],\left[{plx}_{a}\right]\right)$$

The first module, with f_1_ and f_2_, consists of two equations depending on three variables. The last of these, Apc_a_, is the only one belonging to the second module and thus treated as an input parameter. This results in two equations with two variables: for each value of Apc_a_, a solution can be found. With both equations equal to zero, one can reach an expression that determines the first pseudo-nullcline:

$$F\left(\left[{cdk1}_{a}\right],\left[{apc}_{a}\right]\right)=0$$

In the second module, with g_1_ and g_2_, we also have two equations and three variables. The input parameter from the other module is Cdk1_a_. As before, taking both equations equal to zero, one can reach an expression for the second pseudo-nullcline:

$$G\left(\left[{apc}_{a}\right],\left[{cdk1}_{a}\right]\right)=0$$

Finally, we do for both curves a change of variables from Apc_a_ to Cyc_tot_ (total cyclin, the sum of active and inactive complexes), and work in the (Cyc_tot_, Cdk1_a_) phase space (see Supplementary Material for details).

In Figure 1B, we present a scheme of the model following this modular description, as a comparison to the previous scheme based on (Tsai et al., 2014). All parameter values are presented in the Supplementary Material. The parameters changed are reported in the following text and in the Figures.

In Figure 1C, we present the pseudo-nullclines for the system and the corresponding time series trajectory (starting from Cdk1a = 60 nM) for k_synth_ = 0.04, which is just outside the oscillatory range (see Supplementary Material for a bifurcation diagram with k_synth_ as the input, showing two supercritical Hopf bifurcations). The parameter that controls the positive feedback strength, r, is equal to 0.5 (used in the Tsai et al. work). The system trajectory drops and then ascends following the lower branch of C_1_, forming a spiral before ending at a fixed point. The intersection of pseudo-nullclines and the fixed point are within a very small distance of each other, meaning that the intersection represents the stable steady state of the system.

In Figure 1D, k_synth_ = 0.05, which corresponds to a limit cycle of relatively small amplitude. The intersection of curves occurs within the cycle, representing the unstable steady state, and is located just below the fold of C_1_. In a 2D system analyzed with true nullclines, it would be expected for oscillations to occur only when the intersection is located between the two folds of the S-shaped curve. The crossing between our pseudo-nullclines taking place close but below the fold, plus the minimal distance between the intersection and the end point of the time series in the previous case, reflect the “pseudo” character of our method while still showing its usefulness.

In Figure 1E, k_synth_ = 1.5, the value used in the work of Tsai et al. Once again, the trajectory follows the lower branch of C_1_ but not the upper one. This is consistent with results showed by the authors in (Tsai et al., 2008).

Given the bistable shape of the pseudo-nullcline for the Cdk1 module, there is the question of whether both curves could be brought together in a tangential manner. The results shown so far only deal with transversal intersections, with one stable fixed point or limit cycles around an unstable point, born through Hopf bifurcations. A tangency would represent a saddle-node bifurcation, which could act as a SNIC or indicate the existence of a Saddle-Homoclinic (SHom) bifurcation, since there would be a saddle (by virtue of the SN) and a limit cycle (as already established). These global bifurcations would allow more control over the period than what is possible with Hopf bifurcations.

Considering the shape of C_1_, the distance with C_2_, and the composition of Hill functions that goes into C_2_, we performed a few modifications in the model with the goal of bringing about a tangency. First, we added an extra parameter into the differential equation for Apc_a_ (see Supplementary Material). Since low values of Apc_a_ correspond to high values of Cyc_tot_ (outside of the plot scale), adding the extra parameter can bring C_2_ to a drop in Cdk1_a_ close to the right-hand fold of C_1_. At the same time, it could represent basal activity of Apc_a_ in absence of Plx_a_.

**FIGURE 2:**
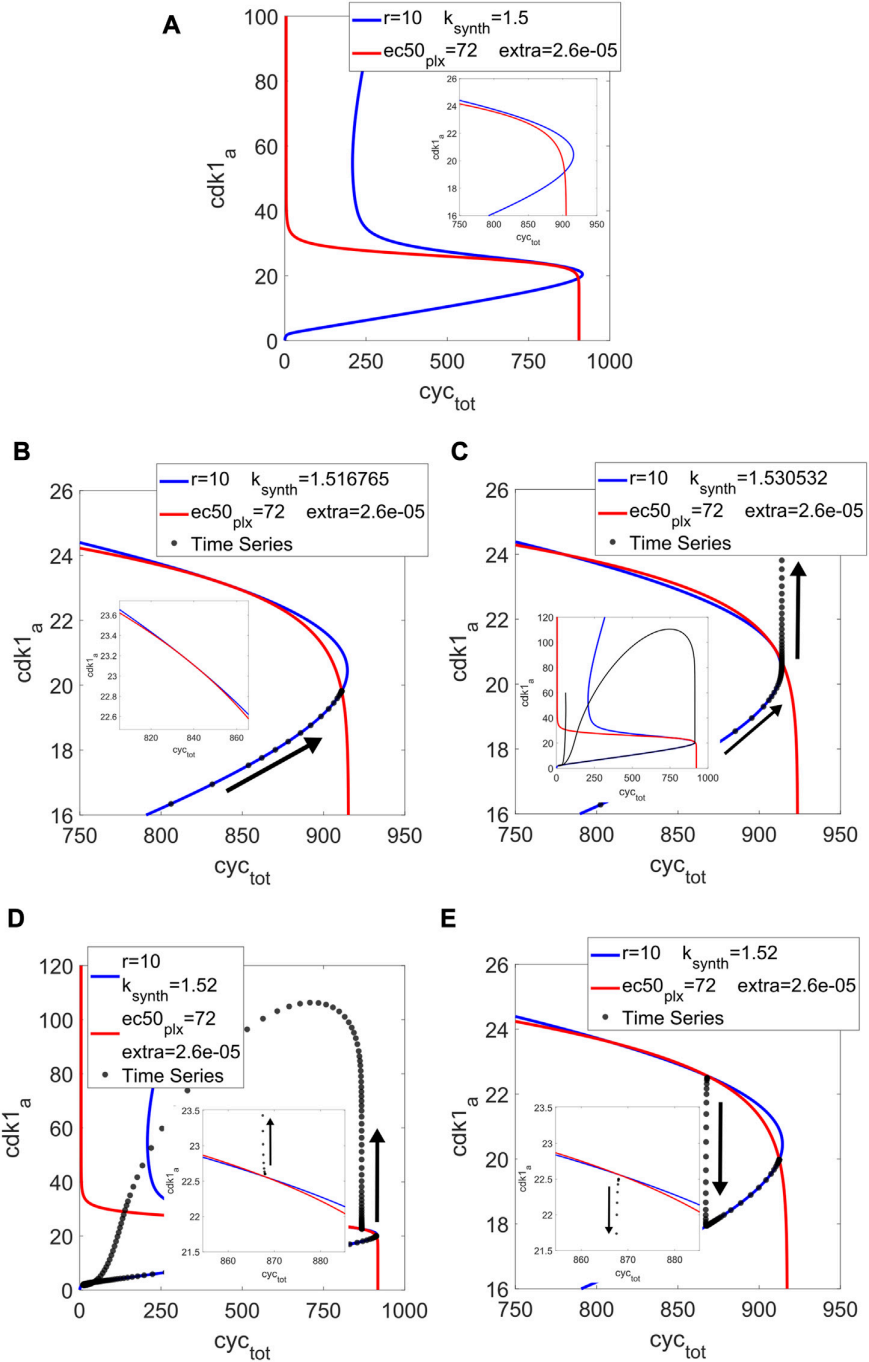
Cell cycle model: pseudo-nullclines C_1_ (in blue) and C_2_ (in red) for r = 10, ec50_plx_ = 72, extra = 2.6e-5 and different input values, with their corresponding time series (in black) for all panels except the first one. **(A)** Input = 1.5, the pseudo-nullclines are close to a tangency, taking advantage of the right-hand fold in C_1_. **(B)** Input = 1.516765, tangency at a distance of the fold, with the time series ending at the lower intersection (the stable steady state). **(C)** Input = 1.530532, tangency close to the fold, intersection between the folds (an unstable steady state), and a limit cycle develops. **(D)** Input = 1.52, taking advantage of the saddle one can choose different initial conditions to obtain excitability (or not). Above the saddle, the system goes around the phase space before ending at the stable steady state. **(E)** Input = 1.52, with an initial condition below the saddle it goes directly to the stable steady state.

With k_synth_ = 1.5, r = 10 (value used in (Tsai et al., 2008)) and increasing ec50 for Plx_a_ to adjust the threshold of C_2_, we arrived at Figure 2A. The distance between the pseudo-nullclines close to the right-hand fold of C_2_ is small enough that a tangency seems possible. We ran the model in MatCont and found two SN at k_synth_ = 1.516765 and 1.530532. Figures 2B,C show the pseudo-nullclines at these values. The tangential behavior of the curves can be appreciated. For the lower input value, the tangency occurs between the C_1_ folds and the transversal intersection outside of them, corresponding to a stable steady state. For the higher input, the tangency is much closer to the C_1_ fold while the intersection is between the folds, representing an unstable fixed point around which the limit cycle takes place. Between these two SN for the full system, an SHom bifurcation was found at k_synth_ = 1.527.

In Figure 2D, we show a case for k_synth_ = 1.52, which is outside of the oscillatory range but between the two SN. Depending on the initial condition, the system can 1) go around the phase space describing one output peak in time before ending at the steady state or 2) take a shorter path to said fixed point. With the initial condition of Figure 2D, just above the saddle point represented by the middle intersection, it goes around. In Figure 2E, it starts from below the saddle, and so it goes directly to lower intersection, corresponding to the stable steady state. The model displays excitability in this region of parameter space, well described by the pseudo-nullclines.

In all, not only our method was consistent with bifurcations born from the original parameter set, but it also allowed us to find a new bifurcation through the manipulation of the two pseudo-nullclines. Moving one parameter at a time facilitates an exploration where the intersections between the curves can change and lead to new findings. In this particular system, the use of Hill functions shows a useful path for the exploration, by modifying the amplitude and threshold of C_2_. We argue that, since Hill functions are prevalent in system biology, this example could serve as inspiration for the analysis of many other cases. At the same time, for any model, the pseudo-nullclines will provide a visual guide for finding new behaviors.

### 3.2 MAP kinase subsystem where both modules are capable of bistability

**FIGURE 3:**
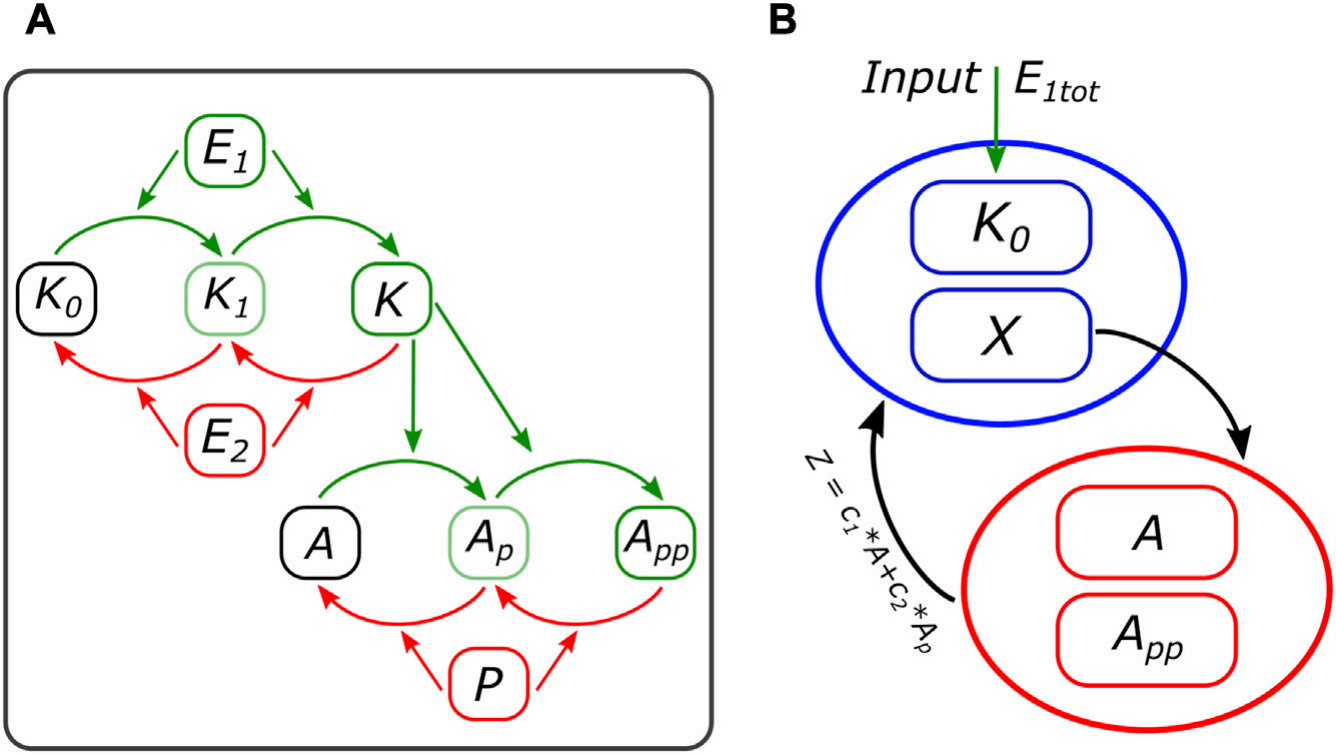
**(A)** Scheme of the 2 + 2 model. The input kinase E_1_ activates the first-level kinase, which goes through two steps before phosphorylating, also in two steps, the second-level substrate. **(B)** Motif scheme based on the pseudo-nullcline method, representing the first- and second-level modules and how they are interconnected through X and Z. More details on the equations can be found in the Supplementary Material.

The second model studied in this work corresponds to the last two levels of the MAPK cascade. It consists of a DP cycle where its output, the double phosphorylated substrate, acts as the kinase for another DP cycle. We will call it the 2 + 2 system, following the double modification process in each level. A scheme is presented in Figure 3A. This motif is of interest for our work, taking the application of the method to a subsystem in an important and well-studied model in biology. But also, there are two important differences with the cell cycle motif from the previous subsection: it is of higher dimension (17 variables versus 4) and capable of bistability in both modules (Markevich et al., 2004).

The parameter set we chose comes from our previous work (Marrone et al., 2023), where the DP cycle displayed bistability when scanning the input kinase. This was a necessary condition to obtain oscillations in the motifs studied and valuable for this work since the presence of oscillations in the model and bistability in each of the two modules (emergent through SN or fold bifurcations) will test the pseudo-nullclines method.

We work with a reduced version of the 2 + 2 system, which can be written as follows (see Supplementary Material for the detailed reduction from the original 17 equations):

$$\frac{d\left[K_{0}\right]}{dt}=f_{1}\left(\left[K_{0}\right],\left[K_{1}\right],X,Z\right)$$

$$\frac{dX}{dt}=f_{2}\left(\left[K_{0}\right],\left[K_{1}\right],X,Z\right)$$

$$\frac{d\left[A\right]}{dt}=g_{1}\left(\left[A\right],\left[A_{p}\right],\left[A_{pp}\right],X,Z\right)$$

$$\frac{d\left[A_{pp}\right]}{dt}=g_{2}\left(\left[A\right],\left[A_{p}\right],\left[A_{pp}\right],X,Z\right)$$

X and Z are functions of some of the original variables:

$$X=\left[K\right]+\left[AK\right]+\left[A_{p}K\right]$$

$${Z=c}_{1}\left[A\right]+c_{2}\left[A_{p}\right]$$

These two are the coupling functions of the model, one for each module, connecting the first and second DP cycle. All parameter values are presented in the Supplementary Material. The input parameter is reported in the following text and in the Figures. Also in the Supplementary Material, a bifurcation diagram for the full system (of 17 equations) with the input E_1tot_ as the parameter, showing four SN bifurcations and two Hopf bifurcations.

**FIGURE 4:**
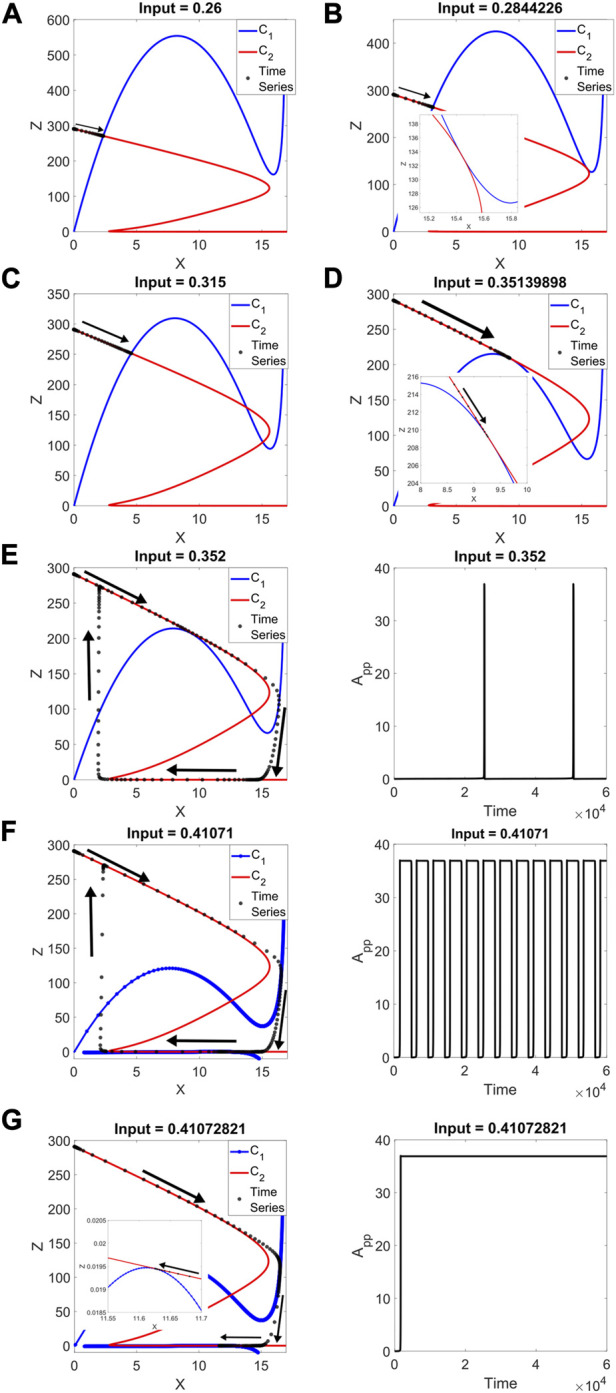
2 + 2 model: pseudo-nullclines C_1_ (in blue) and C_2_ (in red) for different input values, with their corresponding reduced-model time series (in black). Arrows denote the trajectory taken by the system**. (A)** Input = 0.26, only one intersection, where the time series ends. **(B)** Input = 0.2844226, a tangency takes place between the curves, where an SN is located in the full system. **(C)** Input = 0.315, three intersections are found, with the time series going to the representative of the stable fixed point **(D)** Input = 0.35139898, a second tangency for the second full-system SN. **(E)** Input = 0.352. Left: the limit cycle takes full advantage of C_2_’s amplitude. Only one intersection remains, the unstable steady state. Right: the peaks are narrow compared to the time the output is off. **(F)** Input = 0.41071. Left: close to the next SN, the system continues to oscillate with relatively unchanged amplitude, and the first-level pseudo-nullcline shows up for low values of Z (vertical axis plotted from a negative value for clarity). The curves are close to each other. Right: the output is on for a longer time, with brief drops. **(G)** Input = 0.41072821, the tangency at low Z occurs, where the next full-system SN is located. Right: the time series no longer is an oscillation.

In Figure 4A, we show the results at input = 0.26, including the time series for the reduced system. Only one intersection exists, corresponding to a stable fixed point, where the series culminates. It is important to remark once again that both modules are capable of bistability, so it is within the bounds of expectation for both pseudo-nullclines to have folds. In Figure 4B, the input reaches an SN (in the full-system bifurcation diagram), and the two curves are tangent to one another. The new two intersections in Figure 4C represent the new steady states that come after the SN.

In Figure 4D, the input takes the system to a second SN, and the tangency between pseudo-nullclines occurs at a higher value of Z than in Figure 4B. This is coherent with the output A_pp_ being lower on this SN. When Z (combination of A and A_p_) is high, A_pp_ is low and *vice versa*. It is also worth noting that this second tangency takes place near a different fold of the first-level curve.

Starting from this input value, oscillations are found, as shown in Figure 4E. There is only one curve intersection, representing an unstable steady state. This point is located between the two folds of both C_1_ and C_2_. The output spends most of each period at a low level, with brief peaks of activity.

In Figure 4F, the system is close to the next SN. The curves are close to a tangency at a value just above Z = 0. The previous intersection between the folds remains, and two new intersections are close to occur. The output now spends more time at a high level, with relatively brief drops.

The nature of these oscillations comes from the system’s proximity to global bifurcations. When the pseudo-nullclines are almost tangent and the behavior is oscillatory, the trajectory of the system slows down in the vicinity of the almost-tangency. For the input of Figure 4E, the almost-tangency occurs for high Z, low A_pp_. The system can spend a relatively long time in this area. In Figure 4F, at low Z, high A_pp_, the high-level time can be extended with precise manipulations of the input, leaving narrow drops in output.

An interesting aspect of this case is that we have not been able to confirm the presence of SNIC bifurcations via MatCont for the full system (even though SN bifurcations are found when the tangencies occur), while the reduced system cannot be analyzed due to the implicit equations for the conservations (see Supplementary Material). We argue that our method provides further evidence of global bifurcations when a well-known software for analyzing bifurcations falls short of confirmation.

Once the input reaches the next SN, in Figure 4G, the curves are tangent, and the time series stops at that point. At this tangency, the oscillations disappear. The range for stable limit cycles appears limited by two SN bifurcations, with the limit cycle taking advantage of C_2_’s amplitude all along the oscillatory range.

Further scanning of the input shows what is expected, with two new intersections and the time series stopping at the lowest one in Z (the highest in A_pp_). Eventually, the last SN point of the full system is represented by a new tangency close to the left-hand fold of the second-level curve (see Supplementary Material for these last results).

Even though, throughout Figure 4, we are plotting the trajectory of the reduced system, one can find similar results when integrating the full system. And while we cannot obtain with MatCont a bifurcation diagram for the reduced system (as mentioned, due to the implicit nature of the conservation equations), we selected input values following the bifurcations in the full system, with consistent results.

## 4 Discussion

In this work, we applied our pseudo-nullclines method on two models, one corresponding to the embryonic cell cycle and another to a subsystem of the MAPK cascade. They represent two well-known and important examples in systems biology. The parameter sets involved different bifurcations and behaviors, with the purpose of testing the method.

For the Tsai et al. motif, not only we found consistency in our results using the authors’ parameter values, but we were also able to manipulate the pseudo-nullclines toward different bifurcations and therefore, new behaviors. The use of Hill functions for the differential equations was convenient in this regard, and their recurrent use in mathematical modelling of biological systems means that the pseudo-nullclines could be useful for dynamical analysis.

The 2 + 2 motif, unlike the first case, displayed folds for both pseudo-nullclines, representing the underlying bistability in each DP cycle and therefore expanding the pseudo-nullclines application to a bistability-in-both-modules example. The method proved consistent with the motif behavior even though a reduction of the system equations was first necessary, and also helped tracked bifurcations that were not confirmed on MatCont. It remains to be seen whether the method continues to provide useful and consistent results for other regions of parameter space, and how it can be extended to the full MAPK cascade, which involves three modules.

A 2021 work by De Boeck et al. studies the embryonic cell cycle through two bistable switches (a three-equation system), finding high amplitude oscillations with increased robustness: a larger oscillatory region of parameter space than in the case with one bistable switch (De Boeck et al., 2021). Our results, coming from a cell cycle motif (with one bistable module) and a system composed of two bistable modules, could be further developed in this area of cell biology considering the advantages from the work by De Boeck et al. (correct cell cycle progression) and our own (consistent and different behaviors with a four-equation system). In particular, recent work by Parra-Rivas et al. presents a very detailed bifurcation study of various cell cycle models, including the combination of two bistable switches (Parra-Rivas et al., 2023). Our pseudo-nullclines method could be useful for further interpretation in the origin of said bifurcations, which include those of the global type (like the two motifs studied in our present work).

One can find cases in the literature for which our method cannot be applied, like in (Kraikivski et al., 2015) where the system in question, a large cell cycle model in yeast, is divided into a high number of modules, some of them having more than one connection to the rest. It is possible that some type of model reduction or approximation is first necessary to analyze it through pseudo-nullclines. On the other hand, other candidates in the literature are found for applying the pseudo-nullclines method. In (Perez-Carrasco et al., 2018), the authors combine two simple motifs to arrive at a system capable of different behaviors, not obtained with each motif in isolation. The three-equation description is such that two modules are readily determined, each one depending on the other through their coupling variables. The same can be said of the motifs in (Ananthasubramaniam and Herzel, 2014), where the authors lower the degree of cooperativity necessary for oscillations to occur by adding positive feedbacks on three-component negative feedback loops. We believe that the method can be of great value in systems biology, with useful analysis and potential findings in experimental biology.

## Data Availability

The original contributions presented in the study are included in the article/Supplementary Material, further inquiries can be directed to the corresponding authors.
