# Supplementary material for: Pseudo-nullclines enable the analysis and prediction of signaling model dynamics
Source: Front Cell Dev Biol. 2023 Sep 28;11:1209589. doi: 10.3389/fcell.2023.1209589 (PMC10568075; doi:10.3389/fcell.2023.1209589)
Supplement: Supplementary file 1 [file Presentation1.pdf]

Supplementary Material

Tsai et al. 2014 model

It is represented through four differential equations, which are as follows (see Supplementary Material for parameter values):

$$\frac{d[cdk1_a]}{dt} = k_{synth} - k_{dest}[apc_a][cdk1_a] + \frac{1}{\sqrt{r}} k_{cdk1on} \left(1 + p \frac{[cdk1_a]^{ncdc25}}{[cdk1_a]^{ncdc25} + ec50_{cdc25}^{ncdc25}}\right)[cdk1_i] - \sqrt{r} k_{cdk1off} \left(1 + p \frac{ec50_{wee1}^{nwee1}}{[cdk1_a]^{nwee1} + ec50_{wee1}^{nwee1}}\right)[cdk1_a]$$

$$\frac{d[cdk1_i]}{dt} = -k_{dest}[apc_a][cdk1_i] - \frac{1}{\sqrt{r}} k_{cdk1on} \left(1 + p \frac{[cdk1_a]^{ncdc25}}{[cdk1_a]^{ncdc25} + ec50_{cdc25}^{ncdc25}}\right)[cdk1_i] + \sqrt{r} k_{cdk1off} \left(1 + p \frac{ec50_{wee1}^{nwee1}}{[cdk1_a]^{nwee1} + ec50_{wee1}^{nwee1}}\right)[cdk1_a]$$

$$\frac{d[plx_a]}{dt} = k_{plxon} \left(\frac{[cdk1_a]^{nplx}}{[cdk1_a]^{nplx} + ec50_{plx}^{nplx}}\right)(plx_{tot} - [plx_a]) - k_{plxoff}[plx_a]$$

$$\frac{d[apc_a]}{dt} = k_{apcon} \left(\frac{[plx_a]^{napc}}{[plx_a]^{napc} + ec50_{apc}^{napc}}\right)(1 - [apc_a]) - k_{apcoff}[apc_a]$$

Looking at the equations and the scheme, a separation is clear between two modules: 1) the two states (active and inactive) of the Cdk complexes interacting with Cdc25 and Wee1, which form positive feedback loops; 2) Plx_a and Apc_a, which form the negative feedback loop with Cdk1. The first two equations are connected with the last two through Apc_a, which degrades the complexes. On the other hand, Apc_a depends on Plx_a, which in turn depends on $Cdk1_a$.

First, we focus on the two equations for Cdk1. Taking the time derivative for $Cdk1_i$ equal to zero, an expression for $Cdk1_i$ that depends on $Cdk1_a$ and Apc_a can be found:

$$[cdk1_i] = \frac{\sqrt{r}k_{cdk1off}\left(1 + p\frac{ec50_{wee1}^{nwee1}}{[cdk1_a]^{nwee1} + ec50_{wee1}^{nwee1}}\right)[cdk1_a]}{k_{dest}[apc_a] + \frac{1}{\sqrt{r}}k_{cdk1on}\left(1 + p\frac{[cdk1_a]^{ncdc25}}{[cdk1_a]^{ncdc25} + ec50_{cdc25}^{ncdc25}}\right)}$$

Then, with the time derivative for $Cdk1_a$ equal to zero and replacing $Cdk1_i$ with the above, an expression that depends only on $Cdk1_a$ and Apc_a is obtained. By numerically scanning $Cdk1_a$, an implicit equation for Apc_a is left. We solve this with MATLAB, obtaining different pairs (Apc_a, $Cdk1_a$). This is the pseudo-nullcline for the first module, a result of the two differential equations for the Cdk complexes taken at zero.

Now, we apply the same procedure to the Plx_a and Apc_a equations. Taking the time derivative for Plx_a equal to zero, we arrive at:

$$[plx_a] = \frac{k_{plxon}\left(\frac{[cdk1_a]^{nplx}}{[cdk1_a]^{nplx} + ec50_{plx}^{nplx}}\right)plx_{tot}}{k_{plxon}\left(\frac{[cdk1_a]^{nplx}}{[cdk1_a]^{nplx} + ec50_{plx}^{nplx}}\right) + k_{plxoff}}$$

Doing the same for Apc_a:

$$[apc_a] = \frac{k_{apcon}\left(\frac{[plx_a]^{napc}}{[plx_a]^{napc} + ec50_{apc}^{napc}}\right)}{k_{apcon}\left(\frac{[plx_a]^{napc}}{[plx_a]^{napc} + ec50_{apc}^{napc}}\right) + k_{apcoff}}$$

Replacing Plx_a with the above in the equation for Apc_a, we arrive at an explicit equation for Apc_a that depends only on $Cdk1_a$. Once again, we numerically scan $Cdk1_a$, obtaining different pairs (Apc_a, $Cdk1_a$). This is the pseudo-nullcline for the second module, a result of the two differential equations for Plx_a and Apc_a taken at zero. Apc_a and $Cdk1_a$ are the coupling variables of this system.

We take one final step before proceeding. Adding the equations for the Cdk complexes, we obtain:

$$\frac{d([cdk1_a] + [cdk1_i])}{dt} = k_{synth} - k_{dest}[apc_a]([cdk1_a] + [cdk1_i])$$

The sum of the two complexes defines the total cyclin (Cyc_{tot}), with above being its differential equation. When equal to zero:

$$\frac{\boldsymbol{k_{synth}}}{\boldsymbol{k_{dest}[apc_a]}} = [\boldsymbol{cyc_{tot}}]$$

Now, we can perform a change of variables from Apc_a to Cyc_{tot} after arriving at the different pairs (Apc_a, $Cdk1_a$). The variable Cyc_{tot} is the input of the system, and $Cdk1_a$ is the output responsible for mitotic phosphorylation.

<u>Parameter values for the Tsai et. al model, taken from their work (Tsai, Theriot, and Ferrell 2014)</u>:

k_{synth} = 1.5 nM/min

k_{dest} = 0.4

$ec50_{cdc25}$ = 30 nM

n_{cdc25} = 11

$ec50_{wee1}$ = 35 nM

n_{wee1} = 3.5

k_{cdk1on} = 0.0354

$k_{cdk1off}$ = 0.0354

p = 5

$ec50_{plx}$ = 60 nM

$ec50_{apc}$ = 0.5

n_{apc} = 4

n_{plx} = 5

k_{plxon} = 1.5

k_{plxoff} = 0.125

k_{apcon} = 1.5

k_{apcoff} = 0.15

plx_{tot} = 1

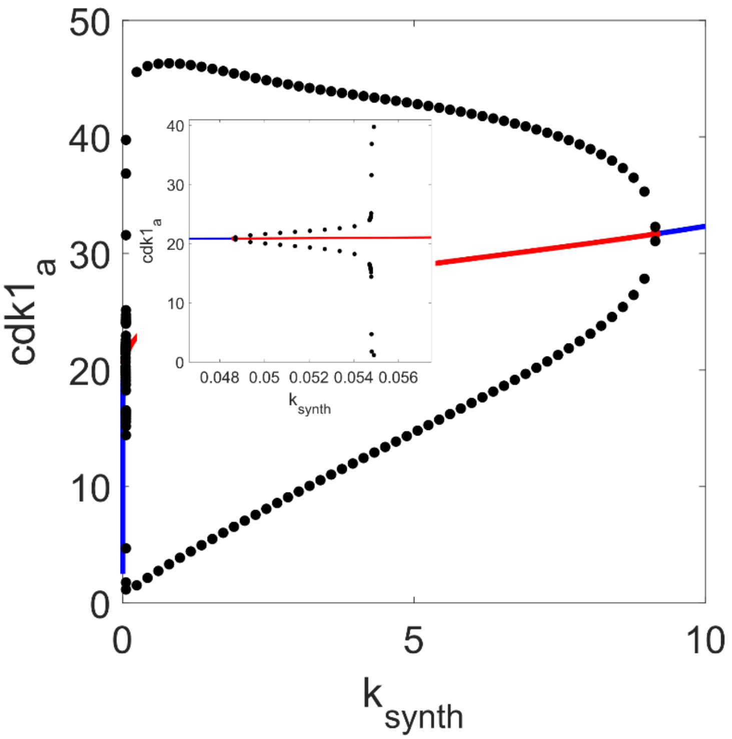

Supplementary Figure 1. One-dimensional bifurcation diagram for the Tsai. et al model with k_{synth} as the parameter, and $cdk1_a$ as the output, using the authors parameter values. Stable steady states in blue, unstable in red, maxima and minima of limit cycles in black circles. Both bifurcations are supercritical Hopf. Near the left-hand Hopf, the amplitude increases rapidly after a short parameter range with small values, as seen in the inset.

For the addition of the extra parameter:

$$\frac{d[apc_a]}{dt} = k_{apcon}\left(\frac{[plx_a]^{napc} + extra}{[plx_a]^{napc} + ec50_{apc}^{napc}}\right)(1 - [apc_a]) - k_{apcoff}[apc_a]$$

And with the differential equation equal to zero:

$$[apc_a] = \frac{k_{apcon}\left(\frac{[plx_a]^{napc} + extra}{[plx_a]^{napc} + ec50_{apc}^{napc}}\right)}{k_{apcon}\left(\frac{[plx_a]^{napc} + extra}{[plx_a]^{napc} + ec50_{apc}^{napc}}\right) + k_{apcoff}}$$

2+2 model

There are 17 coupled differential equations for the 2+2 system:

$$\frac{d[A]}{dt} = k_2[AK] - k_1[A][K] + k_{12}[A_pP],$$

$$\frac{d[AK]}{dt} = k_1[A][K] - (k_2+k_3)[AK],$$

$$\frac{d[A_p]}{dt} = k_3[AK] - k_4[A_p][K] + k_5[A_pK] + k_9[A_{pp}P] + k_{11}[A_pP] - k_{10}[A_p][P],$$

$$\frac{d[A_pK]}{dt} = k_4[A_p][K] - (k_5+k_6)[A_pK],$$

$$\frac{d[A_{pp}]}{dt} = k_6[A_pK] + k_8[A_{pp}P] - k_7[A_{pp}][P],$$

$$\frac{d[A_{pp}P]}{dt} = k_7[A_{pp}][P] - (k_8 + k_9)[A_{pp}P],$$

$$\frac{d[A_pP]}{dt} = k_{10}[A_p][P] - (k_{11} + k_{12})[A_pP],$$

$$\frac{d[P]}{dt} = (k_8 + k_9)[A_{pp}P] - k_7[A_{pp}][P] + (k_{11} + k_{12})[A_pP] - k_{10}[A_p][P]$$

$$\frac{d[K_0]}{dt} = l_2[K_0E_1] - l_1[K_0][E_1] + l_{12}[K_1E_2],$$

$$\frac{d[K_0E_1]}{dt} = l_1[K_0][E_1] - (l_2+l_3)[K_0E_1],$$

$$\frac{d[K_1]}{dt} = l_3[K_0E_1] - l_4[K_1][E_1] + l_5[K_1E_1] + l_9[KE_2] + l_{11}[K_1E_2] - l_{10}[K_1][E_2],$$

$$\frac{d[K_1E_1]}{dt} = l_4[K_1][E_1] - (l_5+l_6)[K_1E_1],$$

$$\frac{d[K]}{dt} = (k_2+k_3)[AK] - k_1[A][K] + (k_5+k_6)[A_pK] - k_4[A_p][K] + l_6[K_1E_1] - l_7[K][E_2] + l_8[KE_2],$$

$$\frac{d[KE_2]}{dt} = l_7[K][E_2] - (l_8 + l_9)[KE_2],$$

$$\frac{d[K_1E_2]}{dt} = l_{10}[K_1][E_2] - (l_{11} + l_{12})[K_1E_2],$$

$$\frac{d[E_2]}{dt} = (l_8 + l_9)[KE_2] - l_7[K][E_2] + (l_{11} + l_{12})[K_1E_2] - l_{10}[K_1][E_2],$$

$$\frac{d[E_1]}{dt} = (l_2+l_3)[K_0E_1] - l_1[K_0][E_1] + (l_5+l_6)[K_1E_1] - l_4[K_1][E_1]$$

The 5 conserved quantities are:

$$E_{1tot} = [E_1] + [K_0E_1] + [K_1E_1]$$

$$E_{2tot} = [E_2] + [KE_2] + [K_1E_2]$$

$$K_{tot} = [K] + [AK] + [A_pK] + [K_0] + [K_0E_1] + [K_1] + [K_1E_1] + [KE_2] + [K_1E_2]$$

$$A_{tot} = [A] + [AK] + [A_p] + [A_pK] + [A_{pp}] + [A_{pp}P] + [A_pP]$$

$$P_{tot} = [P] + [A_pP] + [A_{pp}P]$$

We define X as: $\frac{dX}{dt} = \frac{d[K]}{dt} + \frac{d[AK]}{dt} + \frac{d[A_pK]}{dt}$. The last two terms are zero, following the quasi-steady state approximation. Taking the differential equation for K, one can re-write it as it follows:

$$\frac{d[K]}{dt} = -\frac{d[AK]}{dt} - \frac{d[A_pK]}{dt} + l_6[K_1E_1] - l_7[K][E_2] + l_8[KE_2] + l_9[KE_2] - l_9[KE_2]$$

The last two terms are auxiliary. The section $-l_7[K][E_2] + l_8[KE_2] + l_9[KE_2]$ in equation 8 amounts to $-\frac{d[KE_2]}{dt}$ (quasi-steady state). Taken together, we obtain an equation for X.

$$\frac{dX}{dt} = l_6[K_1E_1] - l_9[KE_2]$$

From quasi-steady state approximation, the differential equations for the first-level intermediate complexes are equal to zero, which leads to:

$$[K_0E_1] = d_1[K_0][E_1],$$

$$[K_1E_1] = d_2[K_1][E_1],$$

$$[KE_2] = d_3[K][E_2],$$

$$[K_1E_2] = d_4[K_1][E_2].$$

Where $d_1 = \frac{l_1}{l_2+l_3}$, $d_2 = \frac{l_4}{l_5+l_6}$, $d_3 = \frac{l_7}{l_8+l_9}$ and $d_4 = \frac{l_{10}}{l_{11}+l_{12}}$. From the conservations for the first-level enzymes E_{1tot} and E_{2tot}, one can write:

$$[E_1] = \frac{E_{1tot}}{1 + d_1[K_0] + d_2[K_1]}.$$

$$[E_2] = \frac{E_{2tot}}{1 + d_3[K] + d_4[K_1]}.$$

The differential equation for X can now be written as:

$$\frac{dX}{dt} = l_6 d_2 [K_1] \frac{E_{1tot}}{1 + d_1[K_0] + d_2[K_1]} - l_9 d_3 [K] \frac{E_{2tot}}{1 + d_3[K] + d_4[K_1]}$$

For the second-level intermediate complexes and applying quasi-steady state, we have $[AK] = c_1[A][K]$ and $[A_pK] = c_2[A_p][K]$. We then have $[K] = \frac{X}{1+c_1[A]+c_2[A_p]}$.

We call $Z = c_1[A] + c_2[A_p]$, which is the coupling function of two second-level variables.

This allows us to replace K in the equation for X:

$$\frac{dX}{dt} = l_6 d_2 [K_1] \frac{E_{1tot}}{1 + d_1[K_0] + d_2[K_1]} - l_9 d_3 X \frac{E_{2tot}}{(1 + Z)(1 + d_4[K_1]) + d_3 X}$$

We apply a similar procedure to $\frac{d[K_0]}{dt} = l_2[K_0E_1] - l_1[K_0][E_1] + l_{12}[K_1E_2]$, where adding $+l_3[K_0E_1] - l_3[K_0E_1]$ forms $-\frac{d[K_0E_1]}{dt}$, which is zero. Then:

$$\frac{d[K_0]}{dt} = l_{12}[K_1E_2] - l_3[K_0E_1]$$

$$= l_{12} d_4 [K_1] \frac{E_{2tot}}{1 + d_3 \frac{X}{1+Z} + d_4[K_1]} - l_3 d_1 [K_0] \frac{E_{1tot}}{1 + d_1[K_0] + d_2[K_1]}.$$

Similar steps are applied to the second level, arriving at two differential equations for A and A_{pp}.

To recap:

$$Z = c_1[A] + c_2[A_p]$$

$$[K] = \frac{X}{1 + Z}$$

The resulting four-dimensional system is:

$$\frac{d[K_0]}{dt} = l_{12}d_4[K_1]\frac{E_{2tot}}{1 + d_3\frac{X}{1+Z} + d_4[K_1]} - l_3d_1[K_0]\frac{E_{1tot}}{1 + d_1[K_0] + d_2[K_1]}$$

$$\frac{dX}{dt} = l_6d_2[K_1]\frac{E_{1tot}}{1 + d_1[K_0] + d_2[K_1]} - l_9d_3X\frac{E_{2tot}}{(1+Z)(1 + d_4[K_1]) + d_3X}$$

$$\frac{d[A]}{dt} = k_{12}c_4[A_p]\frac{P_{tot}}{1 + c_3[A_{pp}] + c_4[A_p]} - k_3c_1[A]\frac{X}{1+Z}$$

$$\frac{d[A_{pp}]}{dt} = k_6c_2[A_p]\frac{X}{1+Z} - k_9c_3[A_{pp}]\frac{P_{tot}}{1 + c_3[A_{pp}] + c_4[A_p]}$$

The two conservations equations are:

$$K_{tot} = [K_0] + [K_1] + X + E_{1tot}\frac{d_1[K_0] + d_2[K_1]}{1 + d_1[K_0] + d_2[K_1]} + E_{2tot}\frac{d_3[K] + d_4[K_1]}{1 + d_3[K] + d_4[K_1]}$$

$$A_{tot} = [A] + [A_p] + [A_{pp}] + X\frac{c_1[A] + c_2[A_p]}{1 + c_1[A] + c_2[A_p]} + P_{tot}\frac{c_3[A_{pp}] + c_4[A_p]}{1 + c_3[A_{pp}] + c_4[A_p]}$$

Setting the first two differential equations to zero, we have the pseudo-nullclines for X and K_0. From each, we arrive at two expressions for Z, the coupling variable. Taking both equal to one another, the following explicit equation for K_0 is obtained (we use the same notation for the variables and their concentrations):

$$K_0 = \frac{l_6d_2K_1E_{1tot}l_{12}d_4K_1 - l_9l_{12}d_4K_1E_{2tot}(1 + d_2K_1)}{l_9E_{2tot}l_{12}d_4K_1d_1 - l_9(1 + d_4K_1)l_3d_1E_{1tot}}$$

With MATLAB, we perform a scan of K_1, obtaining K_0. At this point, two unknown variables remain: X and Z. Using the conservation equation for K_{tot}, replacing each intermediate complex with equations resulting from the quasi-steady state approximation (see above), and replacing Z, an implicit equation for X is reached. After solving it with MATLAB, all that remains is the calculation of Z from either of the previously mentioned expressions.

For the second-level pseudo-nullcline, we rewrite Z as the combination of A and A_p, use the differential equations for A and A_{pp} equal to zero, plus the conservation equation of A_{tot}. This last equation can be written entirely as a function of A_p and A_{pp}. Doing a scan of A_{pp}, we find A_p. Then, we obtain A, which leads to Z and finally to X.

Parameter values for the 2+2 model:

In the first level, every rate constant is equal to 1 with the following exceptions: l_1=8, l_6=50.

In the second level, every rate constant is equal to 1 with the following exceptions: k_1=8, k_3=0.1, k_6=50.

The conserved quantities are E_{2tot}=1, K_{tot}=17, A_{tot}=40, P_{tot}=2.8.

The input E_{1tot} is changed and informed in each result.

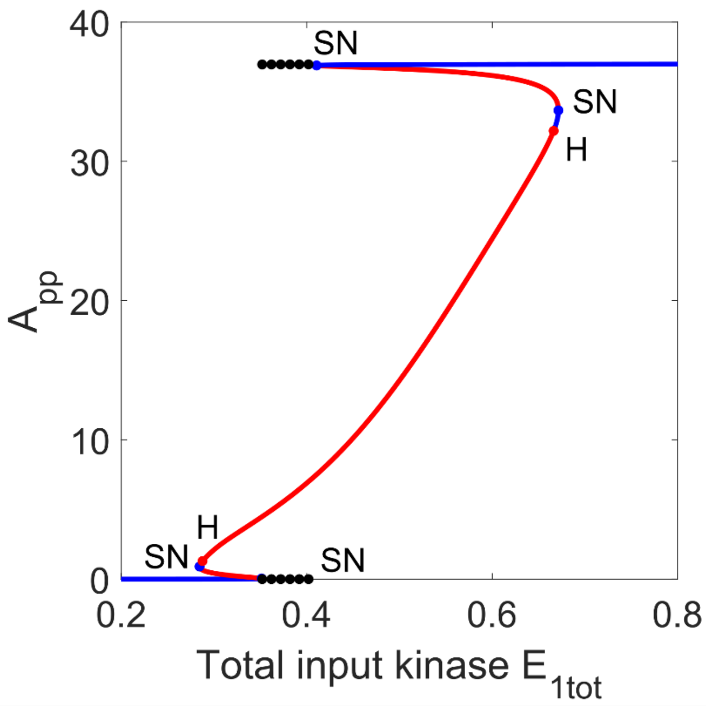

Supplementary Figure 2. One-dimensional bifurcation diagram for the 2+2 model with the total amount of input kinase E_{1tot} as the parameter, and the double-phosphorylated substrate A_{pp} as the output. Stable steady states in blue, unstable in red, maxima and minima of limit cycles in black circles. Very small input ranges with oscillations are found starting from the Hopf bifurcations (H), which we did not consider in the results.

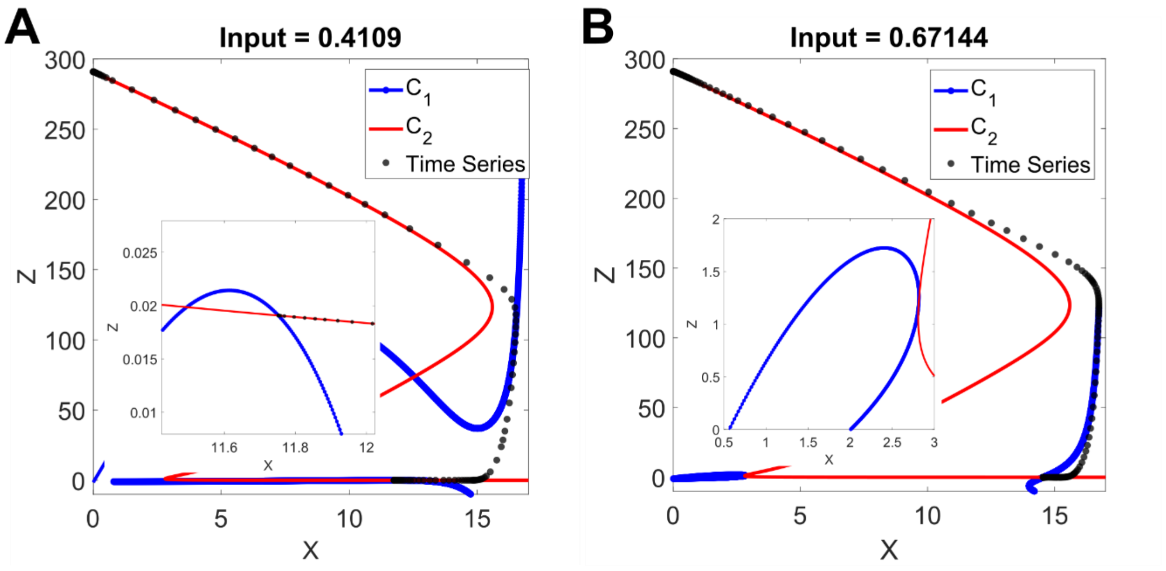

Supplementary Figure 3. 2+2 model: pseudo-nullclines C_1 (in blue) and C_2 (in red) for two different input values, with their corresponding time series (in black). (A) Input=0.4109, three intersections, with the time series ending at the one with the lowest value for Z (highest for A_{pp}). (B) Input=0.67144, a tangency takes place between the curves, where the last full-system SN is located.

References

Tsai, Tony Y.C., Julie A. Theriot, and James E. Ferrell. 2014. "Changes in Oscillatory Dynamics in the Cell Cycle of Early Xenopus Laevis Embryos." *PLoS Biology* 12 (2): e1001788. https://doi.org/10.1371/journal.pbio.1001788.

Supplementary Note

Methods (General description)

The idea of the method is to decompose the system in 2 modules, assuming that the interconnection between the modules is described by 2 one-dimensional functions. This means that if the variables of the modules are denoted respectively by two sets of real variables, i.e. $x = (x_1, x_2, \ldots, x_n)$ and $y = (y_1, y_2, \ldots, y_m)$, the model equations can be written as:

$$
\begin{aligned}
\frac{dx}{dt} &= f(x, \alpha(y)) \\
\frac{dy}{dt} &= g(y, \beta(x))
\end{aligned}
\qquad (1)
$$

where $\alpha(y)$ and $\beta(x)$ are two real-valued functions. Such a system can be seen as a first module, described by equation $\frac{dx}{dt} = f(x,a)$, where a is some input parameter, and $\beta(x)$ is some output function that will connect to the second module. The latter is described by equations $\frac{dy}{dt} = g(y,b)$, where b is the corresponding input parameter, and $\alpha(y)$ the output function. The interconnection between the two modules comes from replacing the input a of the first module by the output function $\alpha(y)$ of the second module, and the input b of the second module by the output $\beta(x)$ of the first module. Such a scheme of decomposing a system into two interconnected modules has been considered in the literature, eg. by [Angeli et al, 2004].

Now, a stationary state (x^*, y^*) of system (1) is a solution of the system of equations:

$$
\begin{aligned}
f(x,a) &= 0 \quad \text{and} \quad a = \alpha(y) \\
g(y,b) &= 0 \quad \text{and} \quad b = \beta(x)
\end{aligned}
$$

Suppose that solutions of the left-handed system of equations can be written as follows:

$$
\begin{aligned}
x &= X(a) \\
y &= Y(b)
\end{aligned}
\qquad (2)
$$

Then, one defines the steady state I/O function of the first module as the function $a \mapsto B(a) = \beta(X(a))$, and likewise the steady state I/O function of the second module as the map $b \mapsto A(b) = \alpha(Y(b))$. When the two modules are coupled, the steady state value of (a^*, b^*) are determined by the two equations:

$$
\begin{aligned}
a^* &= A(b^*) \\
b^* &= B(a^*)
\end{aligned}
\qquad (3)
$$

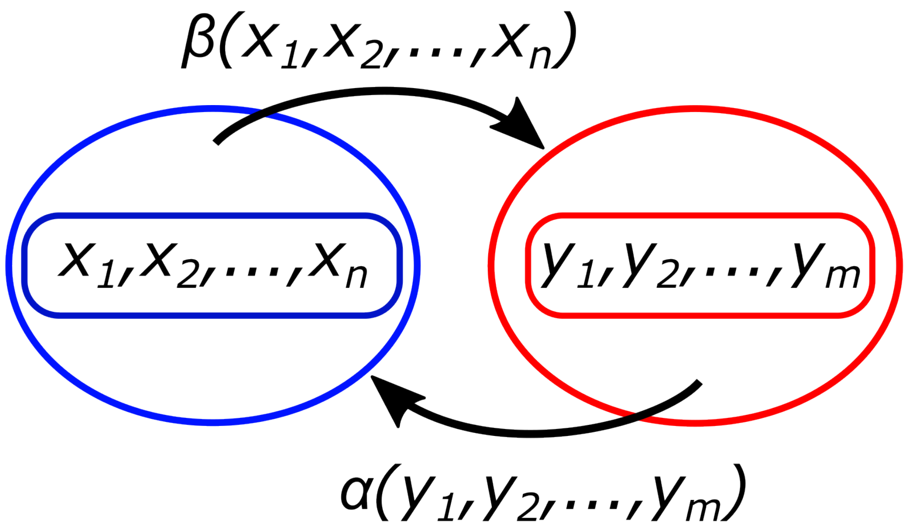

Basic scheme for the method, representing the two modules and their interconnections.

Therefore in the plane of coordinates (a,b), steady states (a^*,b^*) lie in the intersection set of two curves C_1 and C_2 whose graphs are respectively given by the parametrizations $(A(b),b)$ and $(a,B(a))$. This remark motivates the appellation of “pseudo-nullclines" for these curves.
Conversely, if (a^*,b^*) belongs to the intersection of the two curves C_1 and C_2, then $a^* = A(b^*)$ and $b^* = B(a^*)$. Using functions X and Y defined by eqs.(2), one has also that:

$$\begin{aligned} f(X(a^*),a^*) &= 0 \quad \text{and} \quad a^* = \alpha(Y(b^*)) \\ g(Y(b^*),b^*) &= 0 \quad \text{and} \quad b^* = \beta(X(a^*)) \end{aligned}$$

Thus $X(a^*)$ and $Y(b^*)$ constitute a steady state of the coupled modules since the following equations are satisfied:

$$\begin{aligned} f(X(a^*),\alpha(Y(b^*)) &= 0 \\ g(Y(b^*),\beta(X(a^*))) &= 0 \end{aligned}$$

In conclusion the intersections of the pseudo-nullclines C_1 and C_2 are in one-to-one correspondence with the steady states of the coupled modules.
Another advantage of this geometrical method is that it is able to reveal a limit point bifurcation, like a saddle-node bifurcation. As it shown in the S.I., this

occurs when a steady state corresponds to a tangential intersection of the pseudo-nullclines. In particular this feature enables to distinguish between a SNIC bifurcation or a Hopf bifurcation because in the first case oscillations appear through a tangent bifurcation, whereas in the second case the pseudo-nullclines intersect transversely. Both cases are illustrated by applying our method to different signaling motifs studied in the result section.

[Angeli et al, 2004] D. Angeli, J.E. Ferrell, and E. Sontag. PNAS vol. 101, n° 7 (2004) pp. 1822-1827.

Tangency and bifurcation

In this section of the Supplementary Material, we prove the following result: *if a stationary state of the system corresponds to a limit point bifurcation, then the pseudo-nullclines* C_1 *and* C_2 *become tangent at their intersection.*
Referring to the main text, the starting hypothesis is that the stationary states of the system are solutions of the equations:

$$\begin{aligned} f(x,y_1) &= 0 \\ g(y,x_1) &= 0 \end{aligned}$$

where, to fix the ideas, x and f were supposed to be n-dimensional, and y and g to be m-dimensional. Moreover, we assume that a given solution of this system of $n+m$ equations can be written as : $X(y_1)-x=0$ and $Y(x_1)-y=0$. One can consider these two equations as the zero of a unique function $H(x,y)=0$, where H is a $n+m$-dimensional function.
Now, given a stationary state (x^*,y^*), it corresponds to a limit point bifurcation if the derivative $DH(x^*,y^*)$ is not invertible. (Otherwise, by the implicit function theorem, the solution (x^*,y^*) can be uniquely continued, and there is no limit point bifurcation). $DH(x^*,y^*)$ is not invertible if and only if the determinant of the jacobian matrix $DH(x^*,y^*)$ equals zero. Therefore, let us compute the determinant of:

$$DH(x^*,y^*) = \begin{pmatrix} -1 & 0 & \dots & 0 & X_1' & 0 & \dots & 0 \\ 0 & -1 & \dots & 0 & X_2' & 0 & \dots & 0 \\ \dots & \dots & \dots & \dots & \dots & \dots & \dots & \dots \\ 0 & \dots & 0 & -1 & X_n' & 0 & 0 & 0 \\ Y_1' & 0 & \dots & 0 & -1 & 0 & \dots & 0 \\ Y_2' & 0 & \dots & 0 & 0 & -1 & \dots & 0 \\ \dots & \dots & \dots & \dots & \dots & \dots & \dots & \dots \\ Y_m' & 0 & \dots & 0 & 0 & 0 & \dots & -1 \end{pmatrix} \tag{4}$$

The result can be decomposed in 2 terms $A+B$: the first, A, equals to the element DH_{11} (equal to -1) times the corresponding minor. But this minor is the determinant of an upper triangular matrix having only elements -1 on its diagonal. Thus, the result is $A=(-1)^{n+m}$.
The second term, B, can be computed as $DH_{1,n+1}(=X_1')$ times the cofactor $(-1)^n$

times the corresponding minor, that is the determinant of the following matrix:

$$\det\begin{pmatrix} 0 & -1 & \dots & 0 & 0 & \dots & 0 \\ \dots & \dots & \dots & \dots & \dots & \dots & \dots \\ 0 & \dots & 0 & -1 & 0 & 0 & 0 \\ Y_1' & 0 & \dots & 0 & 0 & \dots & 0 \\ Y_2' & 0 & \dots & 0 & -1 & \dots & 0 \\ \dots & \dots & \dots & \dots & \dots & \dots & \dots \\ Y_m' & 0 & \dots & 0 & 0 & \dots & -1 \end{pmatrix} = Y_1'(-1)^{n-1}(-1)^{n+m-4}$$

This result can be obtained by suppressing the line and the column of the element Y_1' (with a cofactor $(-1)^{n-1}$), leaving a $(n+m-4)$ diagonal matrix of -1. Therefore, canceling the determinant of matrix (4) is equivalent to writing:

$$(1-X_1'Y_1')(-1)^{n+m} = 0$$

On the other hand, by construction the pseudo-nullclines C_1 and C_2 are parametrized by $(X_1(y_1), y_1)$ and by $(x_1, Y_1(x_1))$. So, at a given intersection (x_1^*, y_1^*) the components of their tangent vectors can be computed respectively as $(X_1', 1)$ and $(1, Y_1')$. Therefore canceling the determinant:

$$\det\begin{pmatrix} X_1' & 1 \\ 1 & Y_1' \end{pmatrix} = X_1'Y_1' - 1 = 0$$

gives a condition for the alignement of these tangent vectors, and thus for the tangency of the pseudo-nullclines at their intersection.
